# Relations of Dispositions toward Ridicule and Histrionic Self-Presentation with Quantitative and Qualitative Humor Creation Abilities

**DOI:** 10.3389/fpsyg.2018.00078

**Published:** 2018-02-13

**Authors:** Karl-Heinz Renner, Leonie Manthey

**Affiliations:** ^1^Department of Psychology, Bundeswehr University Munich, Munich, Germany; ^2^Department of Psychology, FernUniversität in Hagen, Hagen, Germany

**Keywords:** humor, self-presentation, gelotophobia, gelotophilia, katagelasticism, self-presentation styles, histrionic self-presentation

## Abstract

Previous research has shown that humor and self-presentation are linked in several ways. With regard to individual differences, it turned out that gelotophilia (the joy of being laughed at) and katagelasticism (the joy of laughing at others) are substantially associated with the histrionic self-presentation style that is characterized by performing explicit As-If-behaviors (e.g., irony, parodying others) in everyday interactions. By contrast, gelotophobia (the fear of being laughed at) shows a negative correlation with histrionic self-presentation. In order to further contribute to the nomological network, we have explored whether the three dispositions toward ridicule and laughter as well as histrionic self-presentation are related to humor creation abilities. In doing so, we have assessed the four constructs in a study with 337 participants that also completed the Cartoon Punch line Production Test (CPPT, Köhler and Ruch, 1993, unpublished). In the CPPT, subjects were asked to generate as many funny punch lines as possible for six caption-removed cartoons. The created punch lines were then analyzed with regard to quantitative (e.g., number of punch lines) and qualitative (e.g., wittiness of the punch lines and overall wittiness of the person as evaluated by three independent raters) humor creation abilities. Results show that both gelotophilia and histrionic self-presentation were positively correlated with quantitative and qualitative humor creation abilities. By contrast, gelotophobia showed slightly negative and katagelasticism no associations with the assessed humor creation abilities. These findings especially apply to the subgroup of participants that created punch lines for each of the six cartoons and partly replicate and extend the results of a previous study by Ruch et al. (2009). Altogether, the results of our study show that individual differences in humor-related traits are associated with the quantity and quality of humorous punch lines. It is argued that behavior-related or performative humor creation tasks should be considered in addition to the CPPT in order to open up new avenues that can cross-fertilize research on individual differences in humor and self-presentation.

## Introduction

Since the introduction of gelotophobia, gelotophilia, and katagelasticism as individual differences variables (Ruch and Proyer, 2009a,b), these dispositions toward ridicule and laughter have attracted considerable research activities. Most of the extant studies refer to gelotophobia, the fear of being laughed at (see Ruch et al., 2014 for an overview), that has also stimulated cross-cultural research (e.g., Proyer et al., 2012b; Kamble et al., 2014) and turned out to be a decisive predictor of bullying (Platt et al., 2009). But also gelotophilia, the joy of being laughed at, and katagelasticism, the joy of laughing at others, were investigated in many studies and showed relations, e.g., with character strengths (Proyer et al., 2014), the Big Five (Ruch et al., 2013), parenting styles (Proyer et al., 2012a) in addition to gelotophobia. Furthermore, Renner and Heydasch (2010) have applied a self-presentational view with regard to the three dispositions toward laughter and ridicule. In the remainder of this introduction, we will explicate why self-presentation and especially the histrionic self-presentation style is related to humor and laughter and adds to the nomological network of gelotophobia, gelotophilia, and katagelasticism. Furthermore, we derive hypotheses regarding the relations of the three dispositions toward ridicule and the histrionic self-presentation style with quantitative and qualitative humor creation abilities.

### Gelotophobia, Gelotophilia, Katagelasticism, and Self-Presentation Styles

Self-presentation and humor are linked in several ways (Renner and Heydasch, 2010): First, people need certain presentational skills to create or increase humorous effects. Obviously, some people are more capable in telling jokes and making puns than others. These presentational or performative aspects of joking (e.g., gestures, voice shifts, timing, pantomime) are also referred to as non-verbal humor (Norrick, 2004). Second, self-presentation aims at influencing and managing the impressions of others and this can be achieved by humor and laughter (Rosenfeld et al., 1983), e.g., people can laugh about a joke in order to be perceived as friendly and agreeable, or a young man may amuse his beloved by making jokes in order to enhance his attractiveness (Cooper, 2005). Third, individual differences in self-presentation have turned out to predict wittiness, e.g., high self-monitors according to Snyder (1974), i.e., persons who are skilled and motivated to engage in self-presentation, were assessed as wittier than their low-self-monitoring counterparts in a study by Turner (1980).

Renner and Heydasch (2010) have shown theoretical and empirical links between the three dispositions toward ridicule and laughter with the acquisitive and the protective self-presentation style (Arkin, 1981; Wolfe et al., 1986; Laux and Renner, 2002), and especially the histrionic self-presentation style (Renner et al., 2008).

The first two styles refer to individual differences in self-presentation that are either success- or failure-oriented: Acquisitive Self-Presenters are motivated to adapt their behaviors according to the requirements of a given social situation in order to win social approval. By contrast, protective self-presenters change their behaviors in social situations in order to avoid social disapproval. The theoretical and empirical analysis of the three dispositions toward ridicule in terms of (individual differences in) self-presentation was suggested because Ruch and Proyer (2009a, p. 184) also used a role-theoretical framework in order to explicate gelotophobia, gelotophilia and katagelasticism. Thus, gelotophiles and katagelasticists play rather active roles because they create humor that is directed toward themselves (gelotophilia) or at others’ expense (katagelasticism), whereas gelotophobes play the passive role of being the target of laughter.

It turned out that gelotophobia is markedly associated with the protective self-presentation style that aims at avoiding social disapproval (Renner and Heydasch, 2010). As expected, protective self-presenters tend to interpret being laughed at by others as an indicator of social rejection and disapproval. By contrast, the acquisitive self-presentation style that aims at winning social approval, is negatively correlated with gelotophobia but showed a small positive correlation with gelotophilia. Winning social approval may sometimes be accomplished by making other people laugh at one’s own expense.

The most pronounced positive associations emerged between gelotophilia, katagelasticism and the histrionic self-presentation style, a personality variable that comprises individual differences in using As-If-behaviors in everyday interactions. Histrionic self-presentation is defined “…as a way of shaping everyday interactions by *explicit* As-If-behaviors. Histrionic self-presenters regard daily situations as opportunities for role playing and for transforming such situations into ‘dramatic scenes”’ (Renner et al., 2008, p. 1303). Histrionic As-If-behaviors are not meant seriously and often appear in the form of jokes and teasing. Ironic remarks are subtle forms of As-If-behavior whereas imitating another person by changing one’s voice, mimic, gestures or posture and trying to involve other people in such role plays would be an example of a small dramatic scene. Renner and Heydasch (2010) have pointed out that histrionic As-If-behaviors pervade our everyday life and are often used by entertainers in the media. But even certain politicians sometimes use As-If-behaviors, e.g., the former German minister of defense, Peter Struck, imitated a “Blues Brother” during an election campaign and thus a character from the cult movie of the same name by John Landis.

Based on their theoretical and empirical analyses, Renner and Heydasch (2010) have argued that both dispositions toward ridicule, especially gelotophilia and katagelasticim, and the histrionic self-presentation style may contribute to their respective nomological networks. They suggested that gelotophilia and katagelasticism may be interpreted as specific types of humorous As-If-behaviors that are at the same time associated with different preferences regarding humor appreciation (laughing at oneself with the audience vs. laughing at others). On the other hand, histrionic self-presentation highlights a possible mechanism (doing as-if) that is especially important concerning the performative (or non-verbal) aspects of making jokes about oneself or about others.

### Aims of the Current Research and Hypotheses

In order to further contribute to their nomological networks, we have explored the differential impact of the three dispositions toward laughter and ridicule and histrionic self-presentation with regard to humor creation abilities as assessed in a performance test. As Cronbach and Meehl have already pointed out in 1955, in order to validate a construct that is said to have certain meanings or in more concrete terms in order to “…‘make clear what something is’ …” (Cronbach and Meehl, 1955, p. 290) we need to specify the “laws” in which a construct occurs. Cronbach and Meehl (1955, p. 290) “…refer to the interlocking system of laws which constitute a theory as *a nomological network*” (italics in the original text). Although the term “associative network” seems to be more appropriate in psychology because we seldom can specify real laws but only probabilistic associations, the decisive message is that testable hypotheses may be derived from the proposed meaning or interpretation of a construct. The respective results, then, may or may not contribute to the nomological or associative network and thus to the question what a construct actually “is”.

Ruch et al. (2009) have already shown that gelotophilia and katagelasticism tend to be positively associated with the ability to create humor as assessed with the Cartoon Punch line Production Test (CPPT), in which subjects are asked to write as many witty punch lines to caption-removed cartoons as they can think of. Gelotophobia was unrelated but not negatively associated with humor creation ability as assessed in the CPPT. These results refer to measures of *qualitative* humor creation ability, i.e., the ability to produce punch lines that are evaluated as witty by independent raters and the evaluation of the entire person as witty based on all generated punch lines. Interestingly, neither gelotophilia nor katagelasticism were significantly associated with quantitative humor creation, that is, the number of punch lines created in the CPPT. Gelotophobia even showed a low but not significant negative relation with the quantity of punch lines.

**FIGURE 1:**
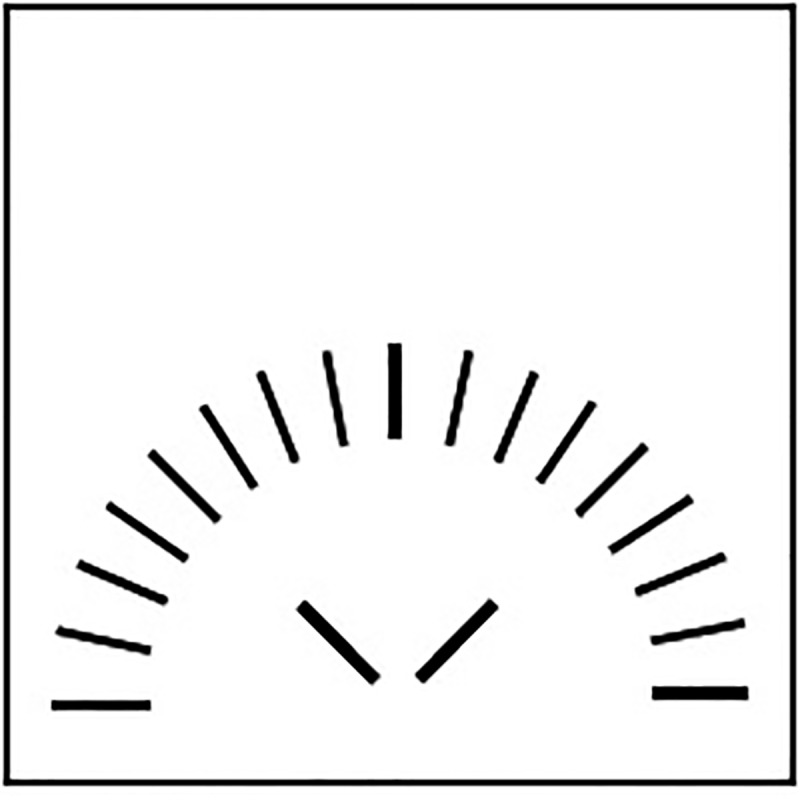
Single-item-originality test.

Up to now, the histrionic self-presentation style was shown to be associated with humor in behavior-related tasks, e.g., the rated humorousness of a presentation task and a simulated talk show in which participants had to play different guests by quickly changing between the respective roles. Histrionic self-presentation was also related to the use of humor as a coping reaction (Renner et al., 2008). Based on these previous findings, one may argue that histrionic self-presenters do need ideas that are at least witty at a medium level as a starting point for their As-If-performances. Indeed, in a single-item originality test, histrionic participants were asked to list as many different and original descriptions as possible for a trivial figure (Renner, 2006, see **Figure 1**).

Here are some of the most original solutions that were determined by a rating procedure:

• Instructions for putting together an IKEA cupboard
• Installation by Josef Beuys: felt rolls made of low fat quark
• Man peeing with a wide range of sprinkling in super slow-motion
• Gymnastics for seniors: Let’s practice lying on our backs

Based on these preliminary findings, we hypothesize that the histrionic self-presentation style should be positively associated with the ability to create witty punch lines in the CPPT. In addition, we also expect a positive relation between histrionic self-presentation and the quantity of punch lines produced. During the course of a spontaneous and improvised histrionic performance the actors need to quickly adapt to the reactions of their interaction partners and generate new witty ideas to be successful. Finally, we wanted to check whether the findings by Ruch et al. (2009) with regard to the humor creation abilities of gelotophobes, gelotophiles, and katagelasticists are replicable.

## Materials and Methods

### Participants and Procedure

This study is based on a total of *N* = 337 participants (254 women) with a mean age of *M* = 33.17 years (*SD* = 10.70, Mdn = 30) ranging from 14 to 63. Participants were first-year undergraduate students of a distance learning program in psychology at a German University (B.Sc.; *N* = 256) and persons from the circle of acquaintances (*N* = 81) of the research group who conducted the data collection of the study. The students of the distance learning university differ regarding age and occupation from the common population of young, mostly female psychology freshmen: Apart from the higher mean for age, the majority of the participants (65,3%) were employed and an additional 21,7% were currently not employed, but had been employed in the past for at least 6 months. Only 13,1% of the sample had never been employed for a period longer than 6 months. Of the 81 participants from the circle of acquaintances of the research group 59 were studying very diverse subjects (9 economics, 6 different teacher training programs, 5 psychology, 4 informatics, 3 educational science, 3 German philology, 2 biology, 2 mathematics and the other 25 participants other specific subjects) at universities and universities for applied sciences throughout Germany.

### Procedure

Participants were invited by email to take part in the study that was conducted completely online using the UNIPARK program of questback. The study was accessible via a link in the emails; this link was also available on the website of the psychology department. Participants had to generate an individual six-digit code according to fixed specifications at the beginning of the study that was used to match their data with other studies. After entering this code, the purpose of the survey and details on data protection were provided. Then, questions on demographic characteristics and the questionnaires on the three dispositions toward laughter and ridicule and the histrionic self-presentation style followed. The quantitative and the qualitative humor creation ability were assessed next using the Cartoon Punch line Production Test (see next section). At the end of each survey, students received a certification of participation.

### Instruments

#### Cartoon Punch Line Production Test (CPPT)

Qualitative and quantitative humor creation abilities were assessed with the German version of the CPPT-K (Köhler and Ruch, 1993, unpublished; Köhler and Ruch, 1996) that consists of six caption-removed cartoons related to the three humor categories incongruity resolution, nonsense, and sexual humor (2 each). These three humor categories were derived from factor-analytic studies and pertain to jokes and cartoons that (1) contain an irritating incongruity that can be completely resolved, (2) contain an incongruity that cannot be or cannot be completely resolved or produces new absurdities (nonsense) and (3) are characterized by more or less explicit sexual content (Ruch, 1992). Although the differentiation between these three categories is important and underlines the background or even “rootedness” of the CPPT in extant humor research, “… it still remains to be shown that the type of humor depicted in the cartoons as well as the contents of the produced responses do indeed matter in the process of humor production (Ruch and Heintz, forthcoming, p. 34). Thus, in the analysis of the punch lines that are produced in the CPPT, the three humor categories are not specifically considered. Ruch and Heintz (forthcoming) report on the validity of the CPPT: It turned out that the CPPT scores were positively correlated with openness to experience and several other self-report measures of humor production, but showed negative associations with seriousness.

Subjects were instructed to create as many punch lines for each of the six cartoons as they were able to. Contrary to the original paper-pencil mode of the CPPT, but similar to the administration of this test in the study by Ruch et al. (2009), we presented the six cartoons online. In accordance with the initial instruction, we administered the CPPT with a time restriction: each cartoon was shown for 2.5 min and then the next cartoon was shown. Thus, each participant produced punch lines in a total period of 15 min.

The total number of punch lines produced by each participant across the six cartoons indicates the *quantitative humor creation ability* (CPPT NP score). Overall, 2771 punch lines were created by the total sample. The number of cartoons for which a punch line was created by each participant (CPPT NC score) may be used as a fluency score. The *qualitative humor creation ability* was assessed by three independent female psychology students who rated the best punch line for each cartoon on a 10-point Likert-scale ranging from 1 = “not witty at all” to 10 = “extremely witty”. Each rater was free to select the punch line she perceived as most witty after reading the created lines for each of the six cartoons. If only one punch line was produced for a cartoon, it was this punch line that was assessed regarding wittiness on the 10-point Likert scale. First, the total score (sum) of the wittiness of the best punch line (averaged across the three raters) for all cartoons (CPPT WP) was calculated. The wittiness ratings were then averaged across the cartoons *for which a punch line was provided* (CPPT WPM score). Thus, this last score is not simply the CPPT WP score divided by six (cartoons) but only divided by the individual number of cartoons for which a punch line was created at all. As a consequence, the CPPT WP score and the CPPT WPM score represent different aspects of the qualitative humor creation ability. Whereas the CPPT WPM score represents the average *maximum* wittiness, the CPPT WP score indicates a combination of fluency and wittiness. The two scores can differ dramatically, e.g., a person who has only created a single punch line that is rated with a high score of, say, 8, will receive this score for both the CPPT WP and the CPPT WPM, whereas another person who has created punch lines for each of the six cartoons that were rated with a score of, say, 6 each, will receive a CPPT WP of 36 and a CPPT WPM of 6.

After the ratings for each punch line were provided, the raters judged the overall wit and fantasy of the person. In general, it is expected that witty persons produce witty punch lines. It may be, however, that also a non-witty person produces a witty punch line once in a while but this exception may not lead to the overall assessment of the person as very witty. The raters were asked how pronounced the ability of a given subject is to produce a witty effect and answered this question on a 10-point Likert scale ranging from 1 = “not at all” to 10 = “extremely strong”. The fantasy of the person was assessed on a bipolar scale ranging from – 4 = “unimaginative” to + 4 = “imaginative” and thus on a 9-point Likert scale (0 is included).

The interrater reliabilities (treating the ratings of the three raters as items) were calculated for each of the six cartoons and the overall wit of the person. The reliabilities for the six cartoons were 0.55, 0.82, 0.83, 0.85, 0.84 and 0.87. Thus, only the reliability for the first cartoon was low and the mean reliability across the six cartoons is still .81. The reliabilities for the overall wit and the fantasy of the person were 0.64 and 0.71, respectively, which may be evaluated as acceptable.

##### PhoPhiKat

The German version of the PhoPhiKat-45 (Ruch and Proyer, 2009a) was administered to assess gelotophobia, gelotophilia, and katagelasticism. The PhoPhiKat-45 measures these three humor-related traits with 15 items per dimension. Ruch and Proyer (2009a) reported internal consistencies of α = 0.88 for gelotophobia, α = 0.87 for gelotophilia, and α = 0.84 for katagelasticism. The respective reliability coefficients in the study at hand were quite similar with α = 0.87 for gelotophobia, α = 0.83 for gelotophilia, and α = 0.83 for katagelasticism. Sample items include “When I have made a fool of myself in front of others I grow completely stiff and lose my ability to behave adequately” (gelotophobia), “For raising laughs I pleasurably make the most out of embarrassments or misfortunes that happen to me which other people would be ashamed of” (gelotophilia) and “Since it is only fun, I do not see any problems in compromising others in a funny way” (katagelasticism). Items are administered with a four-point-scale (1 = strongly disagree; 2 = moderately disagree; 3 = moderately agree; 4 = strongly agree). The validity of the PhoPhiKat-45 was shown in several studies, e.g., in the initial Ruch and Proyer (2009a) study, it is shown that the scores of the three dispositions toward laughter and ridicule were differently related to remembered experiences of being laughed at during childhood. Furthermore, the three dispositions toward laughter and ridicule showed the expected associations with the three dimensions of Eysenck’s PEN model of personality (Eysenck and Eysenck, 1991): Gelotophobia was positively correlated with neuroticism and negatively with extraversion, gelotophilia was primariliy related to extraversion and katagelasticism was positively associated with extraversion and psychoticism (Proyer and Ruch, 2010).

##### As-If-Scale (AIS)

The histrionic self-presentation style was measured by the German version of the As-If-Scale (AIS; Renner et al., 2008). The AIS is an 8-Item scale that covers subtle histrionic forms (“I formulate my statements in such a way that they could have more than one meaning to others”), dramatic performances (“I enjoy putting on a real show for others”), and As-If behaviors that are especially related to changes in body language or nonverbal communication (“When I tell stories I act out the roles of the different participants by imitating their body language and the way they talk.”). The internal consistency was α = 0.82 in this study. The validity of the AIS is shown in Renner et al. (2008): The AIS-score predicted several concrete As-If-behaviors in role-playing tasks and was also associated with the rated wittiness across several role plays. Furthermore, subjects with high scores on the AIS were able two quickly change between different roles in a simulated talk show.

## Results

**Table 1** shows the descriptive statistics and reliabilities for as well as gender differences with regard to the dispositions toward laughter and ridicule and histrionic self-presentation. Means and standard deviations for gelotophobia, gelotophilia, and katagelasticism in our sample were quite similar to those reported by Ruch and Proyer (2009a), and men showed higher katagelasticism than women as well. By contrast, the histrionic self-presentation style was more pronounced in the sample at hand than in most of the previous studies (Renner et al., 2008, study 1, sample 1: *t*(477) = 5.65, *p* < 0.05, *d* = 0.57; Renner and Heydasch, 2010: *t*(978) = 13.09, *p* < 0.05, *d* = 0.86). No differences, however, emerged between the histrionic self-presentation style in the study at hand and study 2 in Renner et al. (2008. *t*(451) = –1.42 n.s.). This result may be due to the fact that both the extant study and study 2 were announced with an explicit hint on humor and As-If behaviors. Thus, self-selection of participants with humorous and histrionic tendencies obviously was the case. As in these previous studies, men scored higher on histrionic self-presentation than women. The skewness and kurtosis statistics show that the distributions of the four traits were reasonably normal.

**Table 1:** Descriptive statistics and gender differences for the dispositions toward laughter and ridicule and histrionic self-presentation.

	Total (*N* = 337)				Women (*N* = 254)	Men (*N* = 83)	Gender differences	
Personality constructs	*M* (*SD*)	*Sk*	*K*	α	*M* (*SD*)	*M* (*SD*)	*t* (*df* = 335)	*d*
Gelotophobia	1.83 (0.51)	0.72	0.12	0.87	1.86 (0.52)	1.76 (0.49)	1.62	0.20
Gelotophilia	2.38 (0.49)	-0.05	-0.01	0.83	2.35 (0.48)	2.47 (0.49)	-1.85^+^	-0.25
Katagelasticism	1.95 (0.46)	0.32	0.13	0.83	1.88 (0.44)	2.17 (0.46)	-5.25^∗∗^	-0.64
Histrionic SPS	2.40 (0.59)	0.59	-0.50	0.82	2.35 (0.58)	2.54 (0.60)	-2.60^∗^	-0.32

*SPS = Self-Presentation Style, Sk = skewness, K = kurtosis, ^+^p < 0.10, ^∗^p < 0.05, ^∗∗^p < 0.01.*

Also, as in previous studies (Ruch and Proyer, 2009a; Renner and Heydasch, 2010), gelotophobia was negatively associated with gelotophilia (*r* = –0.24, *p* < 0.01), whereas gelotophilia was markedly and positively correlated with katagelasticism (*r* = 0.39, *p* < 0.01). As in the study by Renner and Heydasch (2010), but contrary to Ruch and Proyer (2009a), gelotophobia was slightly correlated with katagelasticism in the extant sample (*r* = 0.15, *p* < 0.01). Again (see Renner and Heydasch, 2010), the histrionic self-presentation style showed marked positive associations with gelotophilia (*r* = 0.47, *p* < 0.01) and katagelasticism (*r* = 0.37, *p* < 0.01), but no relation to gelotophobia (*r* = –0.03).

**Table 2** shows the descriptive statistics for the CPPT-scores that indicate quantitative and qualitative humor creation abilities. With regard to the quantitative scores, participants generated an average of approximately 8 punch lines across the six cartoons with a considerable range between 1 and 29 (CPPT NP). Furthermore, punch lines were created for 4 to 5 cartoons on average (CPPT NC), in detail: 40.4% (136 subjects) of the participants created punch lines for the entire six cartoons, 21.7% for five, 17.8% for four, 9.5% for 3, 5.6% for two cartoons and 5.0% for only one cartoon. These quantitative scores were slightly higher than in the study by Ruch et al. (2009) in which the mean for CPPT NP was 7.54 (*SD* = 4.55, range 1–23) and the mean for CPPT NC was 4.36 (*SD* = 1.68, range 1–6).

**Table 2:** Descriptive statistics for CPPT scores that indicate quantitative and qualitative humor creation abilities.

CPPT-scores	*M*	*SD*	*Min*	*Max*	*Sk*	*K*	*Sex*	*Age*
**Quantitative scores**								
CPPT NP	8.22	5.17	1	29	1.12	1.20	0.15^∗∗^	0.15^∗∗^
CPPT NC	4.66	1.47	1	6	-0.97	0.01	0.14^∗∗^	0.21^∗∗^
**Qualitative scores**								
CPPT WP	17.80	7.15	2	39	0.02	-0.36	0.13^∗^	0.09
CPPT WPM	3.83	0.95	1	7	-0.12	0.15	0.04	-0.07
CPPT WI	3.92	1.36	1	8	0.28	-0.13	0.07	-0.03
CPPT FA	-0.24	1.65	-4	4	-0.09	-0.40	0.08	0.00

*Sk = skewness, K = kurtosis, CPPT NP = total number of punch lines, CPPT NC = number of cartoons for which a punch line was created, CPPT WP = sum of the wittiness of the best punch line for all cartoons, CPPT WPM = mean of best punch line across cartoons and raters, CPPT WI = mean wit of the person across raters, CPPT FA = mean fantasy of the person across raters. Correlations with sex and age are non-parametric Spearman correlations. ^∗^p < 0.05, ^∗∗^p < 0.01.*

Concerning the scores that indicate qualitative humor creation abilities, the total score (sum) of the wittiness of the best punch line for all cartoons (CPPT WP) was also higher than in the study by Ruch et al. (2009). The same is true for the mean wittiness of the best punch line across cartoons and raters (CPPT WPM) that is located, however, still below the midpoint of the scale that ranges from 1 to 10. The latter also applies to the wit and the fantasy of the person (CPPT WI). The wit of the person cannot be compared with the respective score in Ruch et al. (2009), because a different scaling (1–7 instead of 1–10) was used in this study and the fantasy scale was not applied.

The skewness and kurtosis statistics show that the distributions of the qualitative scores are reasonably normal. By contrast, the distribution of the total number of punch lines (CPPT NP) is positively skewed and leptokurtic (positive excess kurtosis), i.e., there are few participants that generated punch lines above the average and the distribution shows fatter tails. In addition, several outliers were identified in the distribution of the total number of punch lines. The distribution of the number of cartoons for which a punch line was created (CPPT NC), is negatively skewed and shows a near zero excess kurtosis, i.e., few participants generated punch lines for less than the average number of cartoons. Due to these slight deviations from the normal distribution and especially because of the outliers, we calculated non-parametric Spearman correlations. As these correlations show, age and sex were positively associated with the quantitative indicators of humor creation ability, meaning that male gender and higher age were associated with the creation of more punch lines for more cartoons. In addition, the total score (sum) of the wittiness of the best punch line tended to be higher for men, whereas age showed no significant relation with this indicator of qualitative humor creation ability.

**Table 3** shows the associations between the three dispositions toward laughter and ridicule and histrionic self-presentation with the CPPT-scores. Due to deviations from the normal distribution and outliers with regard to the CPPT, but also the gelotophobia and histrionic self-presentation scores, Spearman rank correlations were calculated as in the study by Ruch et al. (2009). In addition to the analyses in the total sample, we also calculated the correlations separately for the subgroup of participants that were able to create punch lines for each cartoon (i.e., group 6), and for the subgroup of participants that could not provide captions to all cartoons (i.e., group 1–5). This procedure is in accordance with the approach of Ruch et al. (2009) who argued that the group succeeding in generating at least one punch line for each of the six cartoons is of special interest, because one may assume that these participants are characterized by the highest humor production abilities. In addition, one may also argue that only those participants who provided at least one punch line for each of the six cartoons really completed the CPPT.

**Table 3:** Spearman rank correlations between dispositions toward laughter and ridicule, histrionic self-presentation, and the CPPT scores.

CPPT scores	*Gelotophobia*	*Gelotophilia*	*Katagelasticism*	*Histrionic SPS*
**Quantitative scores**				
CPPT NP	0.08	0.11*	0.01	0.10+
CPPT NC	-0.06	0.04	0.02	0.03
**Qualitative scores**				
CPPT WP	-0.10	0.15**	0.04	0.10+
Group 1–5	-0.03	0.07	0.03	-0.01
Group 6	-0.21*	0.33**	0.01	0.22*
CPPT WPM	-0.11*	0.21**	0.04	0.17**
Group 1–5	-0.05	0.12+	0.05	0.14*
Group 6	-0.21**	0.33**	0.01	0.22*
CPPT WI	-0.08	0.17**	0.06	0.14*
Group 1–5	-0.03	0.07	0.06	0.05
Group 6	-0.12	0.30**	0.04	0.27**
CPPT FA	-0.09	0.18**	0.08	0.17**
Group 1–5	-0.07	0.13+	0.11	0.10
Group 6	-0.08	0.25**	–0.02	0.26**

*SPS = Self-Presentation Style, CPPT NP = total number of punch lines, CPPT NC = number of cartoons for which a punch line was created, CPPT WP = sum of the wittiness of the best punch line for all cartoons, CPPT WPM = mean of best punch line across cartoons and raters, CPPT WI = mean wit of the person across raters, CPPT FA = mean fantasy of the person across raters, group 1–5 = 201 participants that created punch lines for 1–5 cartoons, group 6 = 136 participants that created punch lines for each of the 6 cartoons. ^+^p < 0.10, ^∗^p < 0.05, ^∗∗^p < 0.01.*

Concerning the scores that indicate quantitative humor creation abilities (see **Table 3**), the expected positive association emerged between histrionic self-presentation and the total number of punch lines (CPPT NP). The respective correlation, however, was only small and marginally significant, whereas the correlation between gelotophilia and the total number of punch lines was significant at the 0.05-level but also only small. Gelotophobia and Katagelasticism were unrelated to the total number of punch lines and none of the four traits showed associations with the number of cartoons for which a punch line was created (CPPT NC).

Both gelotophilia and histrionic self-presentation showed small correlations with the CPPT scores that indicate qualitative humor creation abilities in the total sample. Thus, high scores for gelotophilia and histrionic self-presentation were associated with more wittiness of the best punch line (CPPT WP and WPM) and a higher degree of the estimated wit and fantasy of the person. These associations, however, were clearly more pronounced and sometimes twice as high as in the total sample in the subgroup that created at least one punch line for each of the six cartoons (group 6 in **Table 3**), and non-existent within the group that only managed to generate punch lines for 1–5 cartoons (group 1–5 in **Table 3**). The respective correlations in group 1–5 and group 6 differ significantly at *p* < 0.05 for gelotophilia with CPPT WP, CPPT WPM, and CPPT WI and for histrionic self-presentation with CPPT WP and CPPT WI. In addition, the correlations with the wittiness of the best punch lines (CPPT WP and WPM) were a little bit higher for gelotophilia than for histrionic self-presentation.

Small negative correlations were found between gelotophobia and the scores indicating qualitative humor creation abilities. However, they were only significant for the mean of the best punch line (CPPT WPM) and for the total score of the best punch line in group 6. No relations were found between katagelasticism and qualitative humor creation abilities.

## Discussion

This study aimed at (1) extending the nomological network of the histrionic self-presentation style by determining associations with humor creation abilities and (2) replicating the results of a former study by Ruch et al. (2009) regarding three dispositions toward ridicule and humor creation abilities. In doing so, relations between gelotophobia, gelotophilia, katagelasticism, and histrionic self-presentation with quantitative and qualitative humor creation abilities as measured in the CPPT were examined. Although the correlation coefficient was only marginally significant, the histrionic self-presentation style was associated with the total number of punch lines created across the six cartoons as hypothesized. Furthermore, histrionic self-presentation was also associated with the wittiness of the best punch lines, as well as the wit and the fantasy of the person. The associations between histrionic-self-presentation and these quantitative and qualitative humor creation abilities were, however, only small to medium. Since it is always more difficult to show associations across different data sources regarding the same or similar constructs, these small to medium correlations may be evaluated as meaningful anyway. Thus, histrionic self-presenters seem to be able to quickly generate many witty ideas that can serve as a starting point for their non-verbal As-If-performances; the witty ideas may be interpreted as a kind of raw material or potential that needs to be elaborated in a concrete interpersonal situation. This potential may be a basic idea for a histrionic role playing game. In order to perform this role playing game, however, the histrionic self-presenter needs to embody it by exhibiting non-verbal As-If-behaviors. In addition, it is also necessary to be responsive to possible interaction partners and consider situational circumstances in order to perform a successful histrionic role playing game.

The most pronounced associations between the three dispositions toward ridicule and laughter with the quantitative and qualitative humor creation abilities as assessed in the CPPT were found for gelotophilia, especially with regard to the subgroup of subjects that managed to create punch lines for each of the six cartoons. The correlations between gelotophilia and the CPPT scores indicating quantitative humor creation ability replicate the findings of the Ruch et al. (2009) study. Since the sample in the study at hand encompasses nearly three times as many subjects as in the Ruch et al. (2009) study, the small correlation between the total number of punch lines with gelotophilia was significant. The correlations between gelotophilia and qualitative humor creation abilities were comparable or higher than in the Ruch et al. (2009) study, especially regarding the six cartoons subgroup. As in the Ruch et al. (2009) study, no associations between gelotophobia and katagelasticism with quantitative humor creation ability were found. Contrary to the findings in Ruch et al. (2009), katagelasticism was unrelated to qualitative humor creation ability even in the group that created punch lines for each of the six cartoons. Thus, in our sample there seem to be only some katagelasticists who are evaluated as witty, whereas others are not. In addition, the relations between gelotophobia and qualitative humor creation ability were slightly negative, especially in the subgroup that created punch lines for each of the six cartoons. Thus, and also in slight contrast to the study by Ruch et al. (2009), the gelotophobes in our sample were evaluated as less witty. In sum, we could only partially replicate the findings of this previous study. In our view, possible reasons for these partially different results in the study at hand are not that much our sample that was in fact bigger but showed similar socio-demographic characteristics. Also, the fact that we only used 3 raters and not 10 as Ruch et al. (2009), did not seem to have a decisive effect in terms of reliability. The most important difference between the two studies was the fact that we used a time restriction - 2.5 min for each cartoon – and Ruch et al. (2009) did not. Research has shown that creative and original solutions usually do need time (e.g., Hennessey and Amabile, 2010). Since there is a strong correlation between originality and wittiness, the time restriction may have impaired the potential for witty solutions at least in gelotophobes and katagelasticists. What argues for this interpretation is the finding that even in the groups that created punch lines for each cartoon, negative or no relations between gelotophobia and katagelasticism, respectively, with qualitative humor creation ability were found. However, this argument does not apply for gelotophiles and histrionic self-presenters, who seem to be superior regarding quantitative and qualitative humor creation abilities even under time pressure. As already argued before, histrionic self-presenters need to quickly generate witty ideas in an ongoing interaction that may be used as a kind of raw material for their histrionic role playing games.

The low to medium correlations between age and sex with the CPPT-scores show that these basic socio-demographic variables have to be taken into account when it comes to predictors of quantitative humor creation ability in particular. Men and subjects with higher age tended to create more punch lines for more cartoons in our study. This result is partly surprising because although men usually show higher humor creation ability (e.g., Mickes et al., 2012) than women, a decline in humor creation ability is assumed with age (e.g., Greengross, 2013). The probably unexpected correlations between age with the total number of punch lines as well as the number of cartoons for which a punch line was created, needs to be qualified with regard to the near-zero correlations of age with the wittiness ratings of the best punch lines. Thus, although older participants produced more punch lines, there seemed to be no significant relation of age with qualitative humor creation ability, i.e., the wittiness of the punch lines.

This study has strengths as well as weaknesses. The results are based on a comparably big and diverse sample and did not only rely on self-report measures but established, partially replicated and extended associations between a cognitive performance task regarding quantitative and qualitative humor creation ability with dispositions toward ridicule and laughter, as well as histrionic self-presentation. Although web-based studies using self-report measures are comparable with paper–pencil studies (Gosling et al., 2004), the same need not be the case with performance tests (Noyes and Garland, 2008): Our participants completed the CPPT online and thus under quite different situational conditions regarding time and place, that might have influenced their performance. Thus, it would have been preferable to administer the CPPT under the same conditions for each participant, e.g., in a big lecture hall with the entire sample. Against the background of research on creativity and originality (Hennessey and Amabile, 2010), the time limit for the generation of witty punch lines could have been a disadvantage regarding the assessment of the maximum humor performance as well. Thus, future studies should administer the CPPT under controlled conditions without time restrictions for each cartoon. In our view, more than six cartoons are not necessarily needed when there are no time limits. Another interesting research question regarding the CPPT would be to determine whether the preferences of the raters for incongruity resolution, nonsense and sexual humor do influence the evaluations of the wittiness of the punch lines. If this obvious hypothesis should be supported, it would be necessary to control for the respective humor preferences of the raters. The same could be considered regarding the raters scores on gelotophobia, gelotophilia, and katagelasticism and possible gelotophobic, gelotophilic, and katagelasticistic contents of the punch lines. Ruch et al. (2009) have already scored the punch lines created in the CPPT regarding these contents and did not find associations with the gelotophobia, gelotophilia, and katagelasticism scores of their subjects. It could, however, well be that the respective scores *of the raters* on these three dispositions toward ridicule influence the ratings of the punch lines that mirror the respective contents.

Further limitations pertain to the sampling procedure in and the gender distribution of our study. As in most psychological studies, we recruited a non-probability self-selection sample, i.e., our sample is neither probabilistic nor representative and thus generalization to whatever population is not possible. In addition, and as already pointed out at the beginning of the results section, participants self-selected to our study that was announced with an explicit hint on humor and as-if behaviors. This self-selection bias and also the fact that much more women than men participated in the study further impede generalization. Thus, further studies with more representative samples that replicate and consolidate the previous findings are necessary.

Future studies should also explore the humor performance of gelotophobes, katagelasticists, and gelotophiles in a behavior-related social interaction task. In doing so, it would be informative to combine a performance test like the CPPT with a task that requires creating humor at the behavioral and interactive level. Thus, in the first part of such a study, participants could be asked to generate as many ideas for shaping an upcoming social interaction as humorous as possible; in the second part, participants could be asked to select the best idea and to perform it together with possible interaction partners. From a self-presentational view, the ability to perform As-If-behaviors especially with gelotophiles and katagelasticists could be determined. From the point of view of dispositions toward ridicule and laughter, it could be explored whether there are gelotophilic and katagelasticistic histrionic self-presenters that exhibit As-If-behaviors aiming at laughing at him or herself together with the audience or at laughing at the expense of others. In doing so, the interactive effects of histrionic self-presentation and gelotophilia and katagelasticism respectively should be determined; e.g., subjects that score high on both histrionic self-presentation and gelotophilia should show the most witty ideas and As-If-behaviors. In addition, the question whether gelotophobes really lack humor could be explored at the behavioral level.

## Conclusion

This study shows that both gelotophilia and histrionic self-presentation are associated with quantitative and qualitative humor creation abilities as measured in the Cartoon Punch line production test, whereas gelotophobia and katagelasticism show slightly negative or no relations regarding humor creation abilities. These findings partly replicate and extend the results of a previous study by Ruch et al. (2009) and open up new avenues that can cross-fertilize research on individual differences in humor and self-presentation.

## Ethics Statement

This study was carried out in accordance with the recommendations and ethical guidelines of the German Psychological Society. All subjects participated anonymously and voluntarily and could quit their participation whenever they wanted without any disadvantages.

## Author Contributions

K-HR and LM planned the study and derived the hypotheses. LM has carried out the entire data collection. K-HR organized the rating procedure with regard to the CPPT, carried out the data analyses and did the writing of the manuscript.

## Conflict of Interest Statement

The authors declare that the research was conducted in the absence of any commercial or financial relationships that could be construed as a potential conflict of interest.
